# Recovery from Unrecognized Sleep Loss Accumulated in Daily Life Improved Mood Regulation *via* Prefrontal Suppression of Amygdala Activity

**DOI:** 10.3389/fneur.2017.00306

**Published:** 2017-06-30

**Authors:** Yuki Motomura, Shingo Kitamura, Kyoko Nakazaki, Kentaro Oba, Ruri Katsunuma, Yuri Terasawa, Akiko Hida, Yoshiya Moriguchi, Kazuo Mishima

**Affiliations:** ^1^(The work was performed in this institution) Department of Psychophysiology, National Institute of Mental Health, National Center of Neurology and Psychiatry, Tokyo, Japan; ^2^Faculty of Design, Kyushu University, Fukuoka, Japan; ^3^Integrative Brain Imaging Center, National Center of Neurology and Psychiatry, Tokyo, Japan; ^4^Division of Medical Neuroimage Analysis, Department of Community Medical Supports, Tohoku Medical Megabank Organization, Tohoku University, Sendai, Japan; ^5^Department of Psychology, Keio University, Yokohama, Japan

**Keywords:** sleep debt, sleep extension, regional cerebral blood flow, arterial spin labeling, functional connectivity, mood, amygdala

## Abstract

Many modern people suffer from sleep debt that has accumulated in everyday life but is not subjectively noticed [potential sleep debt (PSD)]. Our hypothesis for this study was that resolution of PSD through sleep extension optimizes mood regulation by altering the functional connectivity between the amygdala and prefrontal cortex. Fifteen healthy male participants underwent an experiment consisting of a baseline (BL) evaluation followed by two successive interventions, namely, a 9-day sleep extension followed by one night of total sleep deprivation (TSD). Tests performed before and after the interventions included a questionnaire on negative mood and neuroimaging with arterial spin labeling MRI for evaluating regional cerebral blood flow (rCBF) and functional connectivity. Negative mood and amygdala rCBF were significantly reduced after sleep extension compared with BL. The amygdala had a significant negative functional connectivity with the medial prefrontal cortex (FC_amg–MPFC_), and this negative connectivity was greater after sleep extension than at BL. After TSD, these indices reverted to the same level as at BL. An additional path analysis with structural equation modeling showed that the FC_amg–MPFC_ significantly explained the amygdala rCBF and that the amygdala rCBF significantly explained the negative mood. These findings suggest that the use of our sleep extension protocol normalized amygdala activity *via* negative amygdala–MPFC functional connectivity. The resolution of unnoticed PSD may improve mood by enhancing frontal suppression of hyperactivity in the amygdala caused by PSD accumulating in everyday life.

## Introduction

Many modern people are affected, even insidiously, by sleep deprivation in everyday life (1–6). Research has found associations between sleep duration and various health indicators such as obesity (7), depression (8), cardiovascular disease (9, 10), and mortality (11, 12), showing that, while 7–8 h of sleep provides maximum health benefits, any deviation from the duration—either shorter or longer—increases health risks. Unfortunately, many surveys report that ≥30% of the population in advanced countries sleep <6 h (3, 8, 13, 14).

Sleep deprivation that accumulates after having a short sleep over several consecutive days is defined as sleep debt. In a study investigating the effect of different sleep lengths, Van Dongen et al. (15) showed that cognitive performance after 6 h of sleep per night for 2 weeks declined to a level equivalent to that after a night of total sleep deprivation (TSD). The mean sleep duration that the subjects (aged 21–38 years) needed to prevent the accumulation of sleep debt was estimated by the authors to be 8.16 h.

Another interesting finding of Van Dongen et al. (15) was that while task performance continued to decline during the period of sleep deprivation, subjective sleepiness peaked after about 1 week and did not increase thereafter. We normally assess the severity of sleep deprivation using subjective sleepiness as an indicator. Therefore, the dissociation between subjective sleepiness and cognitive function in individuals with chronic sleep deprivation is a risk factor for errors caused by inattentiveness or impaired judgment. In addition, if the individuals could not recognize the presence of sleep debt correctly, they do not make the countermeasure to ensure their optimal sleep duration (OSD), and it may cause prolonged sleep-related problems of them.

These findings suggest that healthy individuals who do not complain about sleep problems or sleep disorder may still have sleep debt, not subjectively in many cases, because of shortened sleep duration in daily life. Indeed, some studies have suggested the existence of sleep debt that stays unnoticed, which is defined as “potential sleep debt” (PSD) (16). When the Multiple Sleep Latency Test (MSLT) was used to objectively assess daytime sleepiness in about 100 healthy young adults, 20–30% of the subjects had a sleep latency of ≤5 min, an indicator of severe sleepiness (17, 18). In a different study using an extended sleep (ES) protocol, subjects without perception of sleep loss during habitual sleep were allowed to sleep as long as needed during a time in bed of 12 h, but they needed such ES for ≥4 days for recovery from PSD (16). Other studies have also shown that sleep extension resulted in improved task performance (19), improved motor skills (20), suppressed excessive appetite (21, 22), and acquired tolerance to partial sleep deprivation (23), suggesting that both body and mind benefit from the resolution of PSD through sleep extension (16).

In addition to decreasing arousal level and cognitive performance, sleep debt also induces emotional dysregulation, including emotional instability such as mood fluctuation and vulnerability to mild stress (24–27) and sympathetic hyperreactivity toward negative emotional stimuli (28, 29). Multiple neuroimaging studies suggest that sleep debt affects the activity of the amygdala, which plays an important role in negative emotional responses (30–34). For example, the medial prefrontal cortex (MPFC) and anterior cingulate cortex (ACC) are thought to have functional and structural connectivity with the amygdala (35), which stabilizes emotions and moods by suppressing amygdala activity (31, 36, 37). When sleep debt is induced after one night of TSD or several nights of a short sleep, the amygdala becomes overactive and the MPFC–amygdala functional connectivity declines, suggesting that a series of these phenomena partly explain the neural basis of anxiety and depressive mood in individuals with sleep debt (30–33). In those studies on emotion, however, the subjects were explicitly forced to be in a sleep-deprived status, which is not likely to reveal their ongoing hidden sleep-related problem, or PSD, in emotional processing in daily life.

We hypothesized that, even without explicit experimental sleep deprivation and even without any awareness of sleep problems or sleep disorders, PSD accumulating in daily life would already have already affected emotional regulation processes in the brain. To test this hypothesis, we recruited normal young adults without any sleep-related problems and measured their mood states (such as anxiety level or and negative affect) as well as resting-state brain activity, focusing on amygdala activity, and amygdala–MPFC connectivity, which reflect emotional regulation processes. Then, in our sleep lab, we let the subjects sleep freely as long as they liked for 9 days to try to resolve their PSD. After their sleep was extended and reached the optimal level, we measured their mood and brain activity and connectivity again. A comparison between the measures before and after sleep extension should reveal the effect of PSD on emotional processing in the brain. After their sleep was optimized, we exposed the subjects to one night of TSD and repeated the measures, which were expected to show the effect of acute sleep loss on emotional processing.

We used arterial spin labeling (ASL) to measure regional cerebral blood flow (rCBF), setting the amygdala and MPFC as regions of interest (ROIs). Resting rCBF correlates with local oxygen consumption, glucose use, and aerobic glycolysis (38, 39) and reflects regional neural activities. Compared with the conventional blood oxygen level-dependent signals, the ASL method robustly resists low-frequency noise and enables the comparison of data from multiple time points because the method is a quantitative measurement of resting-state activity (40). Furthermore, to measure region-to-region functional connectivity, we adopted ASL-functional connectivity analysis (ASL-FC) (40, 41). Combining rCBF with ASL-FC allows us to simultaneously study the magnitude of blood flow in a specific brain area and the functional linkage between that area and other brain regions.

## Materials and Methods

### Ethical Considerations

All subjects gave written informed consent in accordance with the Declaration of Helsinki. The protocol was approved by the Ethics Committee of the National Center of Neurology and Psychiatry (approval number: A2011-071).

### Subjects

As health screening prior to the study, 16 healthy right-handed adult men aged 23.4 ± 2.4 (mean ± SD) years underwent overnight polysomnography (PSG), structural MRI, and hematological testing and completed a questionnaire survey and medical interview with a physician.

Exclusion criteria were as follows: some form of sleep disorder, mental disorder, visual impairment including color blindness, or severe physical complication; current use of medication or substances that might affect the study outcome (e.g., sleep aids, antihistamines, other sleep-inducing drugs, and steroids); a metallic implant such as a pacemaker; travel to a foreign country with a ≥6-h time difference within the last 3 months; shift work; and smoking. One of the 16 subjects dropped out of the study for personal reasons and was therefore excluded from the analysis; thus, all analyses were performed using data from the remaining 15 subjects (aged 23.3 ± 2.1 years). These subjects were originally recruited to study individual PSD (16), and they completed the same interventional protocol. In this article, however, we focused on completely different aspects of the data; that is, we newly adopted a neuroimaging technique to visualize the mood changes and neural responses before and after ES, and the analyses reported here do not overlap those previously published.

### Experimental Protocol

**Figure 1:**
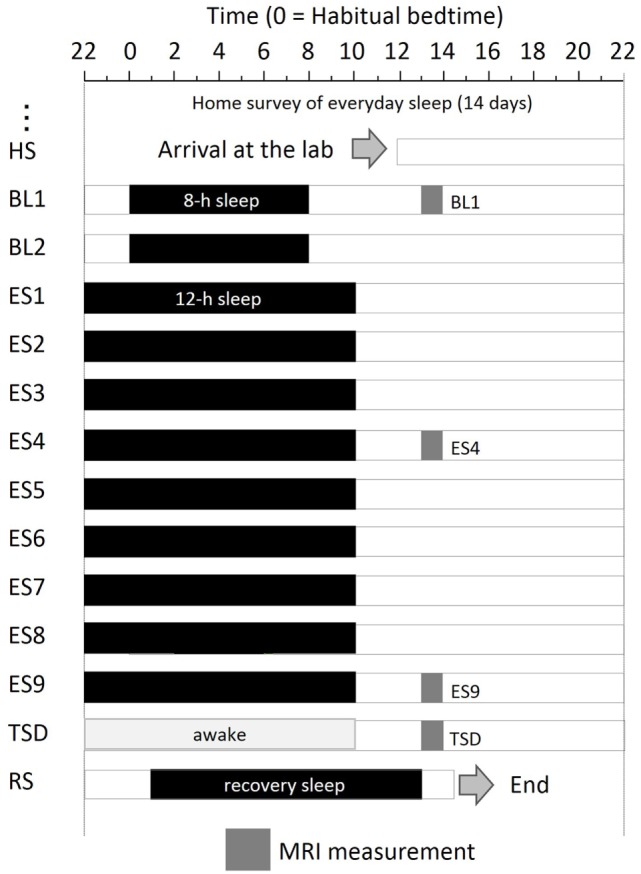
Experimental protocol. Subjects were enrolled in a 14-day and 13-night study, with 8 h of sleep on the first 2 days, 12 h of sleep for the next 9 days, a night of total sleep deprivation, and a night of recovery sleep. MRI was performed at 9 h after the midpoint of sleep on day 1 (BL1), sleep on day 6 (ES4), sleep on day 11 (ES9), and the TSD period on day 12. Subjects underwent overnight PSG measurements throughout the study period. Black bars, sleep time; HS, habitual sleep at home; BL1, baseline day 1; ES4, extended sleep day 4; ES9, extended sleep day 9; TSD, total sleep deprivation; RS, recovery sleep.

The experimental protocol used is shown in Figure 1. After a habitual sleep monitoring period at home (HS period, 2 weeks), the study period consisted of four sessions: a 2-day baseline (BL) period, 9-day ES period, one TSD night, and one recovery sleep (RS) night. On day 1 of the BL period (BL1), on days 4 and 9 of the ES period (ES4 and ES9), and after the TSD, subjective mood was evaluated and ASL MRI was performed.

Subjects recorded bedtime and wake-up time during the HS period. Using the algorithms developed by Sadeh et al. (42), habitual sleep duration (HSD) was calculated from data obtained by a wrist actigraph (Micro-Mini Motionlogger Actigraph, Ambulatory Monitoring, Inc.) worn on the non-dominant hand.

The experiment was conducted in the sleep laboratory at the National Center of Neurology and Psychiatry. This facility includes six isolation units for sleep and a shared living room with electromagnetic shielding for electroencephalography and a homeostatic environment of illuminance, humidity, and temperature. After the HS period, subjects went into the sleep laboratory and were monitored for 14 days. The schedule for each participant was established relative to his at-home records. The average bedtime calculated for each participant was set as 0 (24) h. In the BL period, 8-h time in bed (lights off) started at the mean bedtime observed in the HS period. In the 9-day ES period, bedtime and wake-up time were advanced and delayed, respectively, by 2 h each to make the total time in bed 12 h. At the wake-up time on ES9, TSD began and lasted for 39 h. Then, subjects had RS for 12 h starting 1 h after their habitual bedtime.

During the scheduled wake period, subjects stayed in a living room (approximately 100 lx, 8.7 m × 7.8 m) inside the isolation unit and were allowed to read and write, enjoy music and videos, play videogames, and engage in conversation with a researcher. When falling asleep, subjects were verbally awakened by attendant staff. During the scheduled sleep period, subjects were instructed to sleep in a sleeping room with the lights off (approximately 0 lx) next to the living room and were prohibited from going outside the room except for going to the lavatory. Subjects had three meals per day that met their body mass index and were restricted from unplanned meals and drinks. Ambient temperature and relative humidity in the laboratory were maintained at 25 ± 0.5°C and 50 ± 5%, respectively.

### Polysomnography

Throughout the study period, overnight PSG was performed in a soundproof sleeping room using the Neurofax EEG-1200 system (Nihon Kohden Corporation, Tokyo, Japan) and the following settings: sampling rate of 200 Hz, time constant of hardware high-pass filter of 0.3 s, and hardware low-pass filter of 30 Hz. In addition to electrocardiography, electrooculography, and chin electromyography, PSG data were recorded through Ag-AgCl electrodes placed at Fz, F3, F4, Cz, C3, C4, Pz, O1, and O2, with both earlobes as references, in accordance with the International 10–20 System. Two technicians, who were blinded to the experimental conditions, visually inspected PSG data divided into 30-s epochs and determined sleep stages according to the international standards for sleep classification (43).

Sleep parameters including total sleep time (TST: total time of NREM and REM sleep observed in TIB) were calculated from PSG data obtained every night. The exponential curve was fitted to the TST of all 15 participants, and individual OSD was derived from the lower bound of the curve, as detailed previously (16).

### MRI Task and Questionnaires

Subjective mood was evaluated using the State-Trait Anxiety Inventory-State Anxiety scale (STAI-S) (44) and the Positive and Negative Affect Scale-Negative Affect score (PANAS-NA) (45), and resting ASL imaging was performed 9 h after the midpoint of BL1, ES4, ES9 sleep, and TSD, that is, 5 h after the BL1 wake-up time, 3 h after the ES4 and ES9 wake-up time, and 27 h after the start of TSD.

Subjects had the same breakfast (approximately 350 kcal) 1 h before the MRI and moved into the MRI room. Immediately before imaging analysis, subjects completed the STAI-S and PANAS-NA in the behavioral laboratory next to the MRI room in order to rate current subjective mood. Then, ASL imaging was performed for 4 min. Subjects were instructed to keep their eyes open and look straight ahead to prevent them from sleeping during imaging. We measured subjects’ sleepiness during ASL scanning using the Karolinska Sleepiness Scale (KSS) (46). The KSS consists of a nine-point one-dimensional scale of sleepiness. Immediately after each scan, the subjects were asked about their sleepiness during the scan.

### ASL Scanning Procedure

We obtained ASL imaging between 1800 and 2000 hours on BL1, ES4, ES9, and TSD using a Magnetom Verio 3T MRI System (Siemens). Whole-brain perfusion imaging was performed using a pulsed ASL technique called quantitative imaging of perfusion using a single subtraction, second version with thin-slice TI1 periodic saturation (Q2TIPS). In ASL, arterial blood is magnetically labeled by radiofrequency inversion pulses just below the intracranial area, including the carotid artery. By measuring the difference between the labeled and unlabeled control images, we measured arterial blood perfusion that is not affected by other brain areas and calculated rCBF/100 g/min based on the perfusion value. We obtained 45 time series whole-brain images that were already contrasted (labeled minus unlabeled). The imaging parameters were as follows: repetition time = 2.8 s, echo time = 13 ms, bolus duration (TI1) = 0.7 s, inversion time (TI2) = 2.0 s, number of slices = 12, slice thickness = 8 mm, total labeled scan volume = 45, total unlabeled scan volume = 45, total scan time = 4 min 13 s, and units = mL/100 g/min.

We also obtained a structural image at the time of health screening in each subject using a T1-weighted magnetic resonance rapid gradient-echo (MPRAGE) sequence, with the following parameters: repetition time/echo time = 1,900/2.52 ms, voxel size = 1 mm × 1 mm × 1 mm, flip angle = 9°, and field of view = 256 × 192. The image was used as a spatial reference in the analyses of functional data. Two subjects’ ASL data from ES4 were lost for technical reasons.

### Analyses of rCBF in ASL

Preprocessing and statistical analysis of ASL data were performed using the ASLtbx tool (47) for Statistical Parametric Mapping 12 analysis software (Wellcome Department of Imaging Neuroscience, University College, London, UK^1^). After correction for body movement and slice timing, co-registration to the reference MPRAGE image, and Gaussian smoothing using a kernel of 8 mm full width at half-maximum, rCBF maps were generated by measuring the difference between control and labeled images. Then, the Montreal Neurological Institute template was used to perform spatial normalization. Forty-five rCBF maps thus obtained were averaged to create a mean rCBF map for each imaging analysis.

The ROI was established *a priori*, and the mean intensity within the ROI was calculated for the MRI data obtained at each time point (BL1, ES4, ES9, and TSD). By use of the MarsBaR ROI toolbox for SPM 12, the peak of the ROI (7 mm in radius) was set at the coordinates *x* = 26, *y* = −4, *z* = −20 in the right amygdala and *x* = −20, *y* = −6, *z* = −18 in the left amygdala (Figure 3A). These coordinates were extracted from 639 images stored in the online database at http://Neurosynth.org (48) by using “emotion” as a search keyword. For statistical analysis, the mean blood flow in the voxels in the amygdala ROI was calculated and used in the statistical analyses.

### ASL-Functional Connectivity Analysis

In ASL-FC analysis, a cluster of voxels in the amygdala ROI was set as a seed region bilaterally, and the time series of 45 rCBF maps were analyzed using the CONN toolbox for SPM (Alfonso Nieto-Castanon^2^). A general linear model was applied to the individual time series of signals in each voxel across the whole brain, for four different conditions in each, to estimate a temporal correlation with the rCBF signals in the bilateral amygdala ROIs. The analyses generated ASL-FC beta images representing voxel-wise estimates (correlation coefficients) across the whole brain. To remove noise generated by body movements or physiological processes other than brain activities, the time series of signals of the white matter and cerebrospinal fluid was used as a regressor (the component-based noise correction method aCompCor) (49) and a bandpass filter was applied with the range of 0.008–0.12 Hz. The beta images were entered into the second level, namely, between-subject analyses.

Based on the hypothesis that interaction between the prefrontal cortex and amygdala is involved in mood fluctuations occurring during recovery from sleep debt, the prefrontal cortex was set as the ROI on the ASL-FC beta images under the four different sleep conditions. To extract brain areas with significant connectivity to the amygdala irrespective of the different conditions, the *F* test was performed to analyze connectivities in the beta images created for all four sleep conditions from all subjects (with the two subjects excluded for only ES4 due to loss of data) using the contrast weights vector in the SPM software: [1 0 0 0; 0 1 0 0; 0 0 1 0; 0 0 0 1]. This procedure extracted voxels with functional connectivity significantly larger (positive) or smaller (negative) than 0. Statistical significance was set at *p* < 0.05 (peak level, family wise error corrected) and *k* > 10 contiguous voxels.

The results of the analysis showed that, except for the cluster adjacent to the amygdala, the MPFC was the only brain area with functional connectivity with the amygdala (FC_amg–MPFC_; Figure 4A). The mean value within the cluster in each subject was calculated using MarsBaR, which was used for statistical analysis.

### Structural Equation Modeling

Based on the hypothesis that the amygdala rCBF and FC_amg–MPFC_ explain negative mood during sleep debt (31, 36, 37), we performed multiple regression analyses using a structural equation modeling with all data obtained under the four conditions (*n* = 58) to evaluate the following models: (1) the FC_amg–MPFC_ predicts the amygdala rCBF and the amygdala rCBF predicts the PANAS-NA and STAI-S scores; (2) the amygdala rCBF predicts the FC_amg–MPFC_ and the FC_amg–MPFC_ predicts the PANAS-NA and STAI-S scores; and (3) the amygdala rCBF and the FC_amg–MPFC_ independently predict the PANAS-NA and STAI-S scores. To validate the model fit, we calculated several goodness-of-fit (GFI) indices such as GFI, adjusted goodness-of-fit (AGFI), the comparative fit index (CFI), Tucker–Lewis index (TLI) (50), and Akaike’s information criteria (AIC). The global fit indices were also supported by a GFI > 0.85 (51), an AGFI > 0.80 (52), a CFI > 0.90, and a TLI > 0.90 (51, 53). The AIC is a relative index to compare the multiple model fits, with the smaller value indicating a better fit (54). We modified the model in a stepwise manner based on the Modification Index, which indicates the path after the modification to improve the model fit and effect size, calculated by AMOS software. For each fixed and constrained parameter, the Modification Index is defined as (N/2) times the ratio between the squared first-order derivative and the second-order derivative (55).

### Statistical Analysis

Our research question was twofold, namely, the intervention effects of (1) ES and (2) TSD on participants’ subjective reports on their feelings and their brain activities regarding emotional processes. To investigate the effect of ES, two paired *t* tests, between BL1 and ES4 (with two subjects excluded for only ES4 due to loss of data) and between BL1 and ES9, were performed for each behavior and MRI data with Bonferroni correction (adjusted *p* values (56) are shown). Two paired *t* tests, between BL1 and TSD and between ES9 and TSD, were performed with Bonferroni correction to determine whether the TSD would erase the effect of sleep extension and whether the subjective and neural indices after TSD would be comparable to those at the BL level (i.e., before ES).

Because PANAS-NA scores did not follow a normal distribution in the Kolmogorov–Smirnov test (*p* = 0.20), a non-parametric test (Wilcoxon signed-rank test) was used for an analysis involving the PANAS-NA. Structural equation modeling was performed using SPSS AMOS 23.0.0 and other statistical analyses were performed using SPSS PASW Statistics 18, with the statistical significance set at *p* < 0.05. Descriptive statistics are expressed as means ± SEM.

## Results

### Home and In-Lab Sleep Parameters

On average, the subjects’ in-lab sleep duration at the BL visit did not differ from their in-home HSD. The mean HSD calculated from Actigraph data recorded at home was 7.37 ± 0.27 h, whereas the mean TST calculated from PSG data recorded in the isolation unit on BL1 was 7.35 ± 0.08 h. No significant difference found between the TST on BL1 and the HSD (*t*[14] = 0.048, *p* = 0.962). However, once they were allowed to sleep freely at the beginning of the ES period, subjects began to sleep excessively (>10 h; Table 1), suggesting a compensatory process for the PSD that had accumulated in everyday life. Sleep time gradually reduced with time, approaching their OSD between ES4 and ES9 (16). Their home HSD was significantly shorter than the estimated OSD (HSD = 7.37 ± 0.18 h, est. OSD = 8.41 ± 0.18 h, *t*[14] = 2.858, *p* = 0.0126; Table 1), which suggests the existence of PSD in the participants.

**Table 1:** Average total sleep time (TST) in each experimental day and the optimal sleep duration (OSD).

Day	HSD	BL1	BL2	ES1	ES2	ES3	ES4	ES5	ES6	ES7	ES8	ES9	OSD
Mean (h)	7.37	7.35	7.39	10.59	9.60	9.52	8.96	8.82	8.87	8.63	8.76	8.51	8.41
SEM (h)	0.27	0.08	0.09	0.19	0.28	0.26	0.40	0.23	0.25	0.29	0.17	0.34	0.18

*HSD, average habitual sleep duration measured by actigraphy during a 2-week period before the experiment; TST on each day of baseline (BL) and extended sleep (ES) period was measured by PSG; BL1 and BL2, Day 1 and 2 of the BL; ES1–9, Days 1–9 of the ES; OSD, optimal sleep duration [see our previous publication (16)]; *n* = 15*.

### Questionnaires

**Figure 2:**
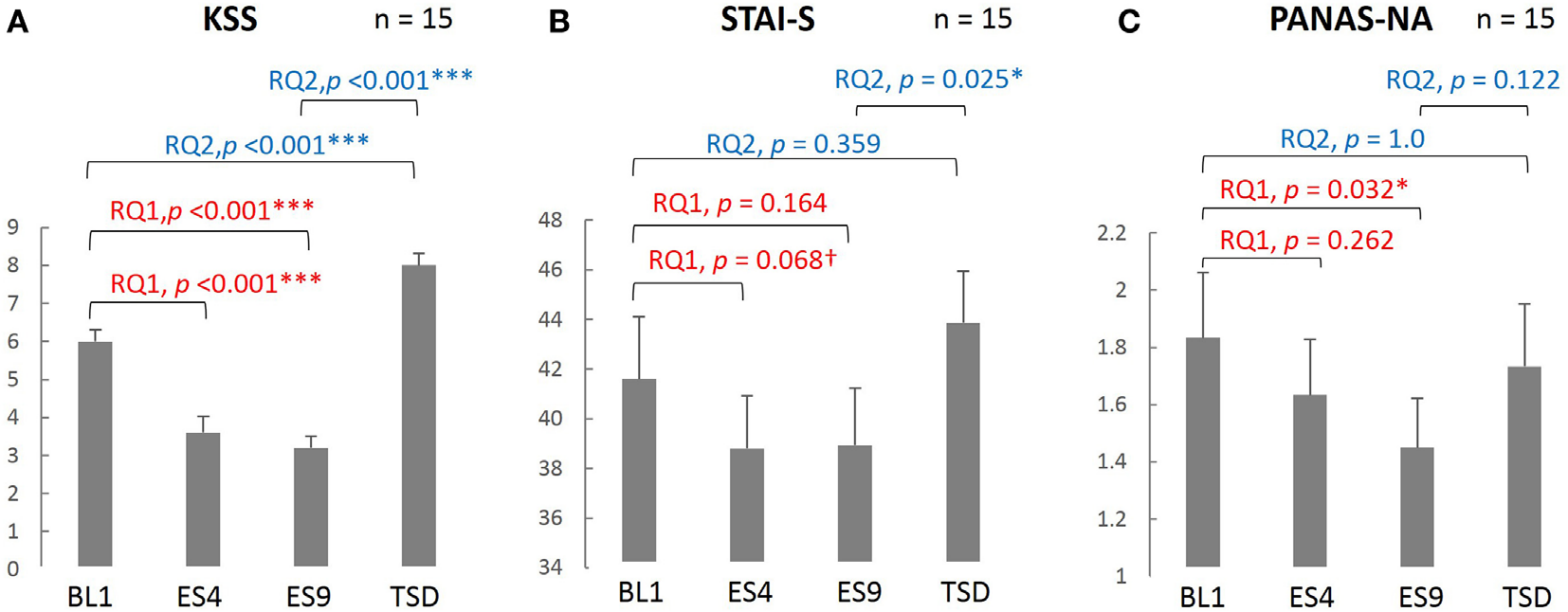
Comparison of the subjective sleepiness and mood state scores among different sleep conditions. KSS **(A)**, PANAS-NA **(B)**, and STAI-S **(C)** were analyzed based on the following two research questions (RQs): (RQ1) Comparison between BL1 and ES9 to investigate the effect of extended sleep (*t* test for KSS and STAI-S and Wilcoxon signed-rank test for PANAS-NA); (RQ2) Comparison between BL1 and TSD to determine whether the TSD would erase the effect of sleep extension and whether the subjective indices after TSD would be comparable to those at the baseline level. ***p < 0.001, *p < 0.05, ^†^*p* < 0.1; *p* values were adjusted for multiple comparison. RQ, research question; KSS, Karolinska Sleepiness Scale; PANAS-NA, Positive And Negative Affect Schedule, Negative Affect; STAI-S, State-Trait Anxiety Inventory-State Anxiety scale; BL1, baseline Day 1; ES4, extended sleep Day 4; ES9, extended sleep Day 9; TSD, total sleep deprivation.

As seen in Figure 2, subjective sleepiness was significantly reduced by sleep extension, from its initial stage: the KSS score was significantly lower on ES4 and ES9 than on BL1 (*t*[14] = 5.527, adjusted *p* < 0.001 and *t*[14] = 6.918, adjusted *p* < 0.001, respectively). Negative affect was also reduced by sleep extension, with a significantly lower PANAS-NA score on ES9 than on BL1 (*Z*[14] = 2.41, adjusted *p* = 0.032), but not in the early stage (no significant difference between ES4 and BL1, *t*[14] = 1.122, adjusted *p* = 0.524). State anxiety showed the same trend, with a marginally lower STAI-S score on ES4 than on BL1 (*t*[14] = 2.349, adjusted *p* = 0.068) and no statistically significant difference between ES9 and BL1 (*t*[14] = 1.874, adjusted *p* = 0.164).

Sleepiness, which had been reduced by sleep extension, increased after TSD (significantly higher KSS score on TSD than on ES9, *t*[14] = 13.052, adjusted *p* < 0.001) and even exceeded the BL level (significantly higher KSS score on TSD than on BL1, *t*[14] = 5.684, adjusted *p* < 0.001). Negative mood, which had been reduced by sleep extension, slightly increased but the change did not reach statistical significance (no significant difference in PANAS-NA scores between TSD and ES9, *Z*[14] = 1.872, adjusted *p* = 0.122), although the negative mood became comparable to the BL level (no significant difference in PANAS-NA scores between TSD and BL1, *Z*[14] = 0.58, adjusted *p* = 1.0). In addition, once-reduced anxiety increased after TSD to a level comparable to the BL: the STAI-S score was significantly higher on TSD than on ES9 (*t*[14] = 2.864, adjusted *p* = 0.025), with no significant difference between TSD and BL1 (*t*[14] = 1.412, adjusted *p* = 0.359).

### ASL rvCBF

**Figure 3:**
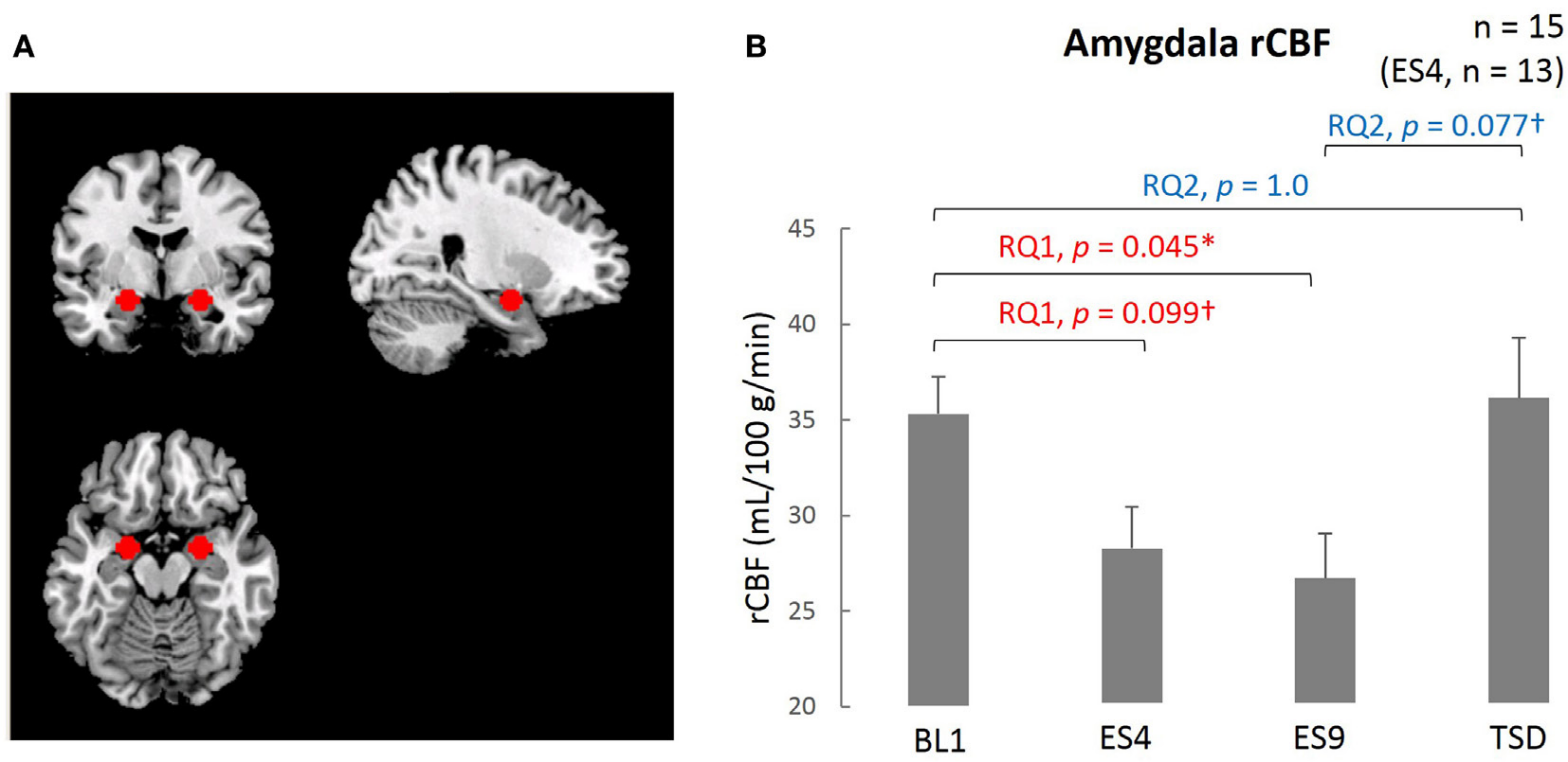
Comparison of the amygdala ROI and rCBF among different sleep conditions. **(A)** Amygdala ROI. Left amygdala: center MNI coordinates (*x, y, z*) = (−20, −4, −16) mm, 179 contiguous voxels; right amygdala, (*x, y, z*) = (24, −4, −16) mm, 178 contiguous voxels; clusters are rendered on a T1 anatomical reference image displayed in neurological convention, with the left side corresponding to the left hemisphere. MNI, Montreal Neurological Institute template. **(B)** Amygdala rCBF for the four sleep conditions. *p < 0.05, ^†^*p* < 0.1; *p* values were adjusted for multiple comparison. Data were analyzed based on the following two research questions: (RQ1) Comparison between BL1 and ES9 to investigate the effect of extended sleep (*t* test for KSS and STAI-S and Wilcoxon signed-rank test for PANAS-NA); (RQ2) Comparison between BL1 and TSD to determine whether the TSD would erase the effect of sleep extension and whether the subjective indices after TSD would be comparable to those at the baseline level. RQ, research question; rCBF, regional cerebral blood flow; BL1, baseline Day 1; ES4, extended sleep Day 4; ES9, extended sleep Day 9; TSD, total sleep deprivation.

The resting-state amygdala activity, shown in Figure 3, was significantly reduced by sleep extension (significantly lower rCBF on ES9 than on BL1, *t*[14] = 2.56, adjusted *p* = 0.046), accompanied by a trend for a reduction in the initial stage (marginally lower rCBF on ES4 than on BL1, *t*[12] = 2.183, adjusted *p* = 0.099). The reduced amygdala activity tended to increase after TSD and became comparable to the BL level (marginally higher rCBF on TSD than ES9, *t*[14] = 2.283, adjusted *p* = 0.077, with no significant difference between TSD and BL1, *t*[14] = 0.248, adjusted *p* = 1.0).

### ASL-Functional Connectivity

**Figure 4:**
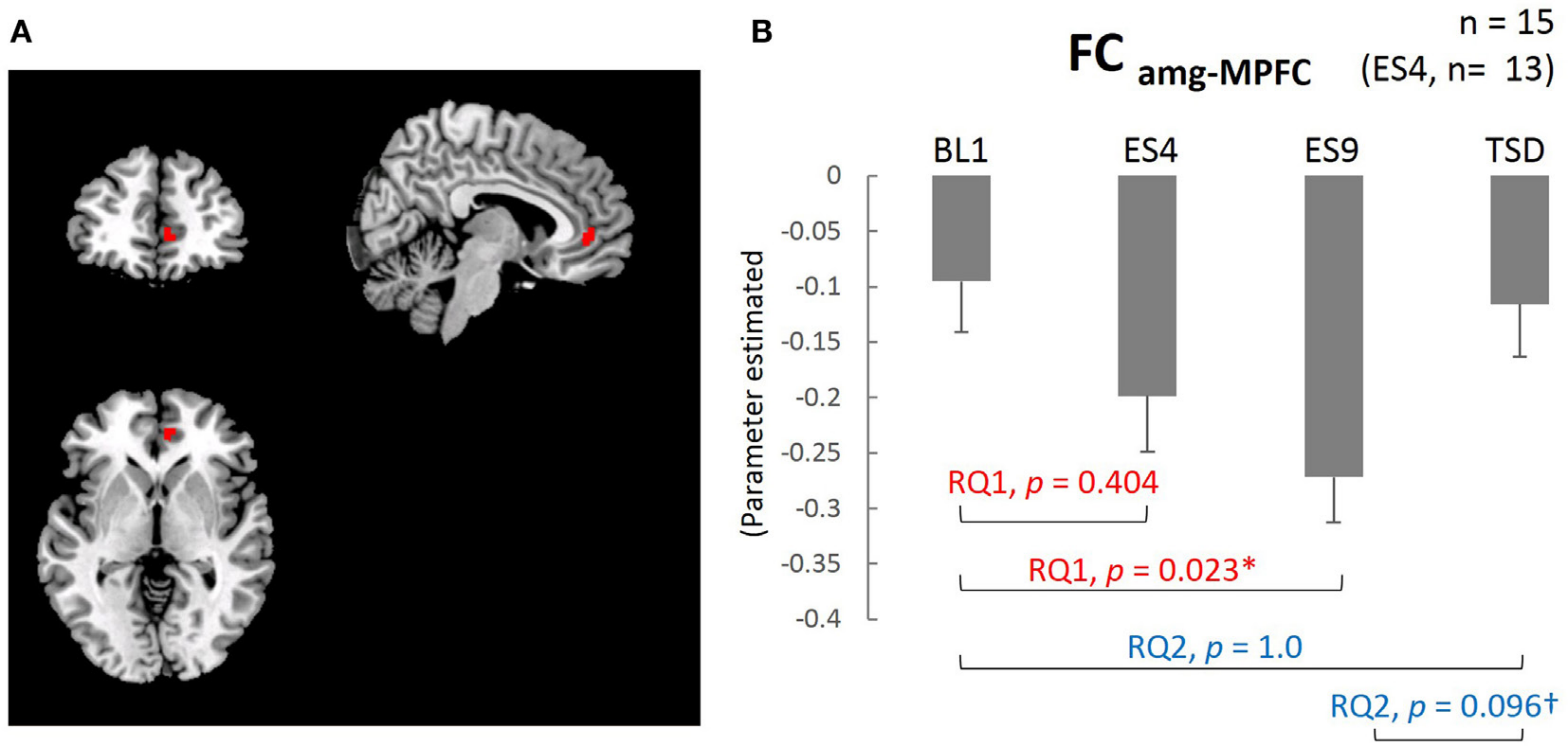
Medial prefrontal cortex (MPFC) ROI and functional connectivity between the amygdala and MPFC. **(A)** MPFC ROI: peak MNI coordinate (*x, y, z*) = (8, 44, 0) mm, 34 contiguous voxels; clusters are rendered on a T1 anatomical reference image displayed in neurological convention, with the left side corresponding to the left hemisphere. MNI, Montreal Neurological Institute template. **(B)** Functional connectivity between the amygdala and MPFC for the four sleep conditions. *p < 0.05, ^†^*p* < 0.1; *p* values were adjusted for multiple comparison. Data were analyzed based on the following two research questions: (RQ1) Comparison between BL1 and ES9 to investigate the effect of extended sleep (*t* test for KSS and STAI-S and Wilcoxon signed-rank test for PANAS-NA); (RQ2) Comparison between BL1 and TSD to determine whether the TSD would erase the effect of sleep extension and whether the subjective indices after TSD would be comparable to those at the baseline level. RQ, research question; FC_amg–MPFC_, functional connectivity between the amygdala and medial prefrontal cortex; BL1, baseline Day 1; ES4, extended sleep Day 4; ES9, extended sleep Day 9; TSD, total sleep deprivation.

The extent of negative functional connectivity between the amygdala and MPFC, shown in Figure 4, was increased by sleep extension, but not from the earlier stage of the sleep extension (larger FC_amg–MPFC_ on ES9 than on BL1, *t*[14] = 2.905, adjusted *p* = 0.023, with no significant difference between ES4 and BL1, *t*[12] = 1.35, adjusted *p* = 0.404). The increased negative connectivity then showed a trend for a decrease after TSD and became comparable to the BL level (marginally lower FC_amg–MPFC_ on TSD than on ES9, *t*[14] = 2.165, adjusted *p* = 0.096, with no significant difference between TSD and BL1, *t*[14] = 0.301, adjusted *p* = 1.0).

### Structural Equation Modeling

**Figure 5:**
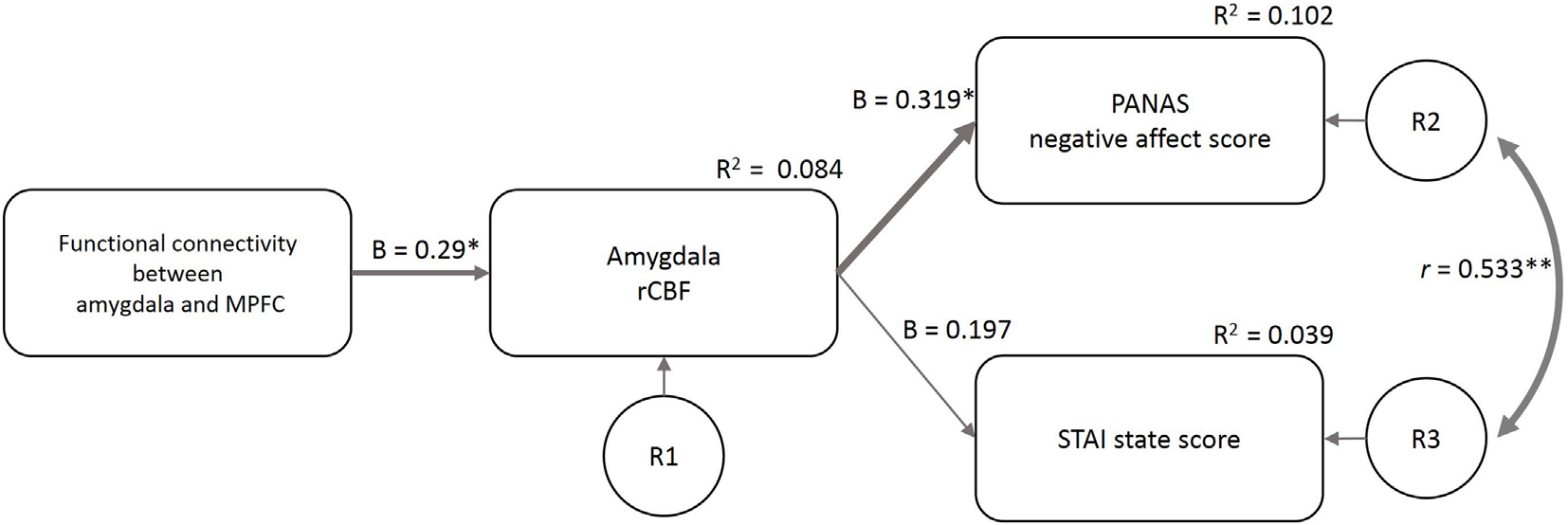
Structural equation modeling of psychological and neuroimaging variables. The functional connectivity between the amygdala and MPFC predicted the amygdala rCBF, which further predicted the PANAS-NA and STAI-S scores. The model included the data of the four sleep conditions (*n* = 58). β, standardized coefficient; *R*^2^, square of multiple correlation coefficient; *r*, correlation coefficient; R1–3, residual of each endogenous variable; FC_amg–MPFC_, functional connectivity between the amygdala and medial prefrontal cortex; rCBF, regional cerebral blood flow; PANAS-NA, Positive And Negative Affect Schedule-Negative Affect score; STAI, State-Trait Anxiety Inventory-State Anxiety score. ^†^*p* < 0.1, *p < 0.05, **p < 0.01.

The fit indices at the first estimate of the three models supported *model 1* (GFI = 0.86, AGFI = 0.54, CFI = 0.35, TLI = −0.3, AIC = 36.32) more than *model 2* (GFI = 0.80, AGFI = 0.35, CFI = 0.09, TLI = −0.82, AIC = 44.04) and *model 3* (GFI = 0.87, AGFI = −0.30, CFI = 0.35, TLI = −2.89, AIC = 38.25). Based on the Modification Index, we added a path between the residuals for the PANAS-NA and STAI-S to *model 1* (considering the correlation between the two mood indices) and made the final model (Figure 5). GFI indices strongly supported the final model (GFI = 0.97, AGFI = 0.87, CFI = 0.96, TLI = 0.88, AIC = 19.24). In this model, the FC_amg–MPFC_ explained the variance in the amygdala rCBF (β = 0.29, *p* = 0.022), and the amygdala rCBF explained the variance in the PANAS-NA score (β = 0.319, *p* = 0.011). The amygdala rCBF did not explain the variance in the STAI-S (β = 0.197, *p* = 0.129).

## Discussion

Over the 9-day ES period in this study, sleep duration increased significantly from that recorded at home prior to the study, but the duration decreased gradually with time to attain stable levels, namely, OSD (16). Furthermore, the OSD was longer than the HSD at home, and subjective sleepiness ratings decreased after sleep extension. These findings showed that sleep debt that had possibly accumulated in everyday life was resolved by sleep extension.

As expected, the negative affect levels (measured by the PANAS) improved after the sleep extension but reverted to the same level as at BL due to the subsequent TSD. The magnitude of the PSD (estimated as OSD − HSD) was approximately 1 h per day in this study, which was mild compared with the amount of sleep debt caused by forced sleep deprivation in previous reports (30, 31, 33) (around 3–4 h per day). Nevertheless, this mild PSD induced a robust decline in mood, the extent of which was identical to the level of mood decline induced by a night of TSD. This result suggests that PSD is non-negligible in terms of its impact on emotional processing, all the more because it is scarcely recognized consciously by individuals.

Arterial spin labeling analysis revealed that amygdala rCBF decreased after the resolution of PSD but increased again after the TSD, in parallel with the negative affect measured by the PANAS. In our path analysis, the amygdala rCBF predicted the PANAS-NA. Elevated resting amygdala rCBF is strongly associated with negative emotional responses and psychiatric symptoms such as depression (57, 58). Although accumulating evidence shows that total or partial sleep deprivation increases amygdala activity toward negative emotional stimuli (30, 31, 33), what we have shown for the first time is that even PSD accumulating every day, not a forced experimental sleep deprivation, had already inflated the amygdala rCBF, which could be normalized by a sleep extension protocol. Taken together, the results of our study may suggest that PSD could be a risk factor for psychiatric emotional problems including depression, considering the short sleep duration prevalent among modern people suggested by epidemiological studies (8).

In our present study, the STAI and PANAS-NA, indicators of subjective mood, showed different trends in changes during the sleep extension and TSD (Figure 2): although the STAI-S was relatively responsive from the earlier stage of sleep extension as well as after the acute TSD, the PANAS-NA changed gradually throughout the sleep extension to reach a significant change in the later stage; however, its response to the TSD was not statistically significant. The two measures also differed in their correlation with the amygdala rCBF, such that the amygdala rCBF predicted the PANAS-NA but not the STAI-S. Although the two questionnaires similarly detect negative mood, they measure different aspects of emotional states: the STAI is specifically focused on anxiety and the PANAS-NA measures more holistic negative emotions, including more social or situational emotions such as “hostile,” “guilty,” or “ashamed.” Transient anxiety measured by the STAI-S may possibly respond quickly to a forceful stress (such as sleep deprivation), whereas general negative affects measured by the PANAS-NA may be responsive to relatively slower and milder interventions (such as recovery from sleep debt). Indeed, the STAI-S score increased after a 5-day partial sleep deprivation in our previous study (34), and the STAI-S responded to an acute anxiety-inducing task, unlike the PANAS-NA (59). Given that amygdala activity and connectivity also showed gradual changes during sleep extension, we can speculate that a gradual recovery from PSD has an ameliorating effect on a broader spectrum of negative affects, not specific to anxiety.

Along with the changes in amygdala rCBF, we observed a significant increase in the extent of negative FC_amg–MPFC_ on ES9, which reverted to the level of the BL1 after TSD. Note that in our path analysis, there was a correlation between the magnitude of the negative FC_amg–MPFC_ and the reduction in the amygdala rCBF, which successively explained the variance in the PANAS-NA score. Such a neural path that we present here is supposed to reflect the suppression of the amygdala by the MPFC, which is the core mechanism of emotional regulation. In contrast, previous neuroimaging studies have shown the involvement of *positive* FC_amg–MPFC_ in the association between experimentally induced sleep debt and emotional regulation (30–34). However, it may not be unnatural to see such a discrepancy between studies, considering the detailed mechanism of emotional regulation that occurs in the interaction between the ACC/MPFC and amygdala. Growing evidence suggests the existence of a “feedback circuit” between the amygdala and ACC or medial prefrontal area, which is highly involved in emotion regulation (60). In the circuit, during the presentation of threatening stimuli, the rostral subgenual ACC (rACC) has a positive connectivity with the amygdala, whereas the caudal ACC (cACC) has a negative one. This means that we can observe not only positive but also negative connectivity between the amygdala and ACC/MPFC, probably because different studies would spotlight different parts of affective processes that recruit a variety of components in this feedback circuitry; animal studies in the primate brain have shown two-way neural projections, from the amygdala to the rACC and from the cACC to the amygdala (61). Such looping connectivities (amygdala-rACC-cACC-amygdala) were hypothesized to organize a feedback circuitry that regulates neural processing in the amygdala in the face of some environmental adversity. In our study, the ACC/MPFC area that showed significant *negative* functional connectivity was located closer to the cACC than the area with functional connectivity in previous studies (30–34). Taken together, the *negative* amygdala-ACC/MPFC connectivity observed in our study might reflect the latter part of the feedback circuitry, namely, more regulatory function of the cACC on the amygdala, and this suppressive function might be more vulnerable to one’s stressed sleep status, such as PSD. In contrast to our focus on sleep extension, which was expected to regain regulation of amygdala hyperactivity, the experimental acute sleep deprivation adopted in the previous studies should focus more on exacerbating the overactivation in the amygdala, which might involve a change in the former part of the circuit (i.e., amygdala-rACC), resulting in more emphasis on *positive* functional connectivity. Because of the limitation of this study that the sample size is relatively small, this consideration is however still speculative and warrants further study.

## Conclusion

Our findings suggest that healthy adults suffer from PSD at a subconscious level, which adversely affects mood regulation. Mood can be regulated efficiently, however, by resolving the sleep debt and thus normalizing the inhibitory function (presumably functional connectivity) of the frontal region to suppress the amygdala hyperreactivity. It is suggested that sleep extension suppresses amygdala activity and improves mood by enhancing prefrontal suppression of the amygdala.

## Ethics Statement

All subjects gave written informed consent in accordance with the Declaration of Helsinki. The protocol was approved by the Ethics Committee of the National Center of Neurology and Psychiatry (approval number: A2011-071).

## Author Contributions

YM, SK, AH, YoM, and KM conceived and designed the experiments. YM, SK, KN, KO, RK, YT, and AH performed the experiment. YM, SK, and KN analyzed the data. YM, SK, YoM, and KM wrote the main manuscript text. All authors reviewed the manuscript.

## Conflict of Interest Statement

The authors declare that the research was conducted in the absence of any commercial or financial relationships that could be construed as a potential conflict of interest.

## Footnotes

^1^http://www.fil.ion.ucl.ac.uk/spm/software/spm12/.

^2^http://www.alfnie.com/software/conn.
